# Classical trajectories in polar-asymmetric laser fields: Synchronous THz and XUV emission

**DOI:** 10.1038/srep34973

**Published:** 2016-10-19

**Authors:** Aram Gragossian, Denis V. Seletskiy, Mansoor Sheik-Bahae

**Affiliations:** 1 University of New Mexico, Physics and Astronomy Dept., 1919 Lomas Blvd. NE, Albuquerque, NM 87131, USA; 2 Department of Physics and Center for Applied Photonics, University of Konstanz, 78457 Konstanz, Germany

## Abstract

The interaction of intense near- and mid-infrared laser pulses with rare gases has produced bursts of radiation with spectral content extending into the extreme ultraviolet and soft x-ray region of electromagnetic spectrum. On the other end of the spectrum, laser-driven gas plasmas has been shown to produce coherent sub-harmonic optical waveforms, covering from terahertz (THz) to mid- and near-infrared frequency spectral band. Both processes can be enhanced via a combination of a driving field and its second harmonic. Despite this striking similarity, only limited experimental and theoretical attempts have been made to address these two regimes simultaneously. Here we present systematic experiments and a unifying picture of these processes, based on our extension of the semi-classical three-step model. Further understanding of the generation and coherent control of time-synchronized transients with photon energies from meV to 1 keV can lead to numerous technological advances and to an intriguing possibilities of ultra-broadband investigations into complex condensed matter systems.

Generation and manipulation of coherent radiation at extreme wavelengths has advanced immensely in the recent years. Significant progress has been made in the field of high harmonic generation (HHG) where researchers routinely produce bursts of extreme ultraviolet (XUV)^1^,^2^ and soft x-ray^3^,^4^ radiation by focusing intense near-infrared laser pulses into rare gas targets. Along with the advent of novel diagnostic schemes^5^,^6^,^7^, these advancements have led to the generation of isolated sub-100 attosecond pulses^8^,^9^ and can potentially support zeptosecond pulses in the near future^4^. At the same time, great progress has been made in the generation and detection of intense optical waveforms covering from sub −1 THz to beyond 100 THz frequency range^10^,^11^,^12^,^13^,^14^. Broadband electromagnetic pulses in both of these spectral extremes can be efficiently generated and manipulated using a two-color excitation scheme where the fundamental driving pulse is co-focused with its second harmonic (SH) onto a rare gas plasma^15^,^16^,^17^.

The generation of high harmonics from rare gases follows a non-perturbative mechanism, with its salient features captured by a classical three-step model^18^,^19^. In this picture, electrons are liberated in a tunneling ionization process and accelerated every half cycle of the driving laser pulse. Depending on their birth time, a fraction of these electrons can acquire sufficient kinetic energy to trigger pulsed emission of high-frequency photons upon recombination with the parent ion. Taking into account polar symmetry of the multi-cycle laser field and the centro-symmetry of the gaseous media, the resulting XUV pulse train contains only odd harmonics of the carrier frequency ω_0_. Injection of a small fraction of second harmonic (SH) field facilitates breaking of the polar symmetry enabling emission of odd as well as even harmonics^15^,^20^,^21^. Information on the time of birth of the high frequency emission^20^,^22^,^23^ is encoded in the spectral structure of the high harmonics, thus providing a useful route for gating single attosecond pulses^9^.

The presence of second harmonic drastically enhances emission in the THz band. Similar to the HHG, the THz generation mechanism is described semi-classically including tunneling ionization of electrons followed by their classical response to an oscillating optical field^24^, mimicking the first two steps of Corkum’s three-step model. Those electrons that do not return to the parent ion may instead create a transient net current which can support bandwidth in excess of 100 THz when excited with few-optical cycle pulses^25^. Therefore, it is interesting to note that coherent electromagnetic transients in the extreme frequency range spanning from THz to XUV can be produced by essentially the same method of optical two-color excitation while sharing similar origin in the physical description (Fig. 1). The intriguing similarities in the physics of HHG and THz generation together with the practical implications of temporal synchronization provide motivation for understanding their correlations and coherent control.

In recent years, various groups have investigated the spectral features of high harmonic generation under *weak* polar asymmetry in two-color excitation^20^,^22^,^26^. Additionally, there has been an investigation of simultaneous THz and XUV emission under similar conditions. Most of the above data have been reconciled qualitatively with the prediction of the semi-classical strong-field-approximation (SFA). In this paper, we extend the experimental scope to include the regime of strong polar asymmetry and investigate HHG correlation from short as well as long electron trajectories with the synchronous THz emission. Most importantly, we present a simple and intuitive formalism that extends the Corkum’s three-step model. By analyzing the classical “closed” electron trajectories, we are able to explain all the salient features observed not only in our data but all the previously published experiments on high harmonic generation in gasses by two-color fields. Instead, for “open” trajectories the XUV yield with strong asymmetry is predicted to be minimized and instead being replaced by THz radiation, thus recovering the results of the plasma current model^24^. More generally, by treating the two processes on an equal footing we are able to establish the correlations between the XUV and THz emission mechanisms and use this information to shed further light onto the generation of XUV in the cases of weak-polar asymmetric pulses.

Simultaneous generation of XUV and THz radiation is investigated by varying polar asymmetry of a co-polarized two-color excitation by adjusting the ratio (*η*) of the second harmonic to the fundamental field from weak *η* = *0.005* (*0.5%*) to strong *η* = *0.1* (*10%*) (see Methods section). For a fixed ratio of *ω*_*0*_ and *2ω*_*0*_ fields, asymmetry can also be continuously modulated by changing the relative phase *δϕ* between these phase-locked constituents. We demonstrate that the underlying complexity of the response can only be observed when both *η* and *δϕ* are varied systematically. In comparison, earlier attempts to draw conclusions on the underpinning relationship between THz and XUV generation mechanisms were considered only in the regime of weak SH injection, resulting in case-specific observations, which depended on the particular values of the observed order of the high harmonics^27^,^28^.

To elucidate the intricate nature of the highly nonlinear interaction of matter with such designer waveforms, we conducted measurements of simultaneous THz and XUV emission from argon gas (Fig. 1) as a function of *δϕ* for strong (*η* = 0.1, Fig. 2(a,b)) and weak (*η* = 0.005, Fig. 2(c–f)) polar asymmetry in the driving field. As expected, in both of these cases the emission shows a characteristic oscillatory dependence on the sub-cycle polar asymmetry of the driving field, controlled by *δϕ.* First we concentrate on the details of simultaneous emission for *η* = 0.1. Fig. 2(a,b) shows the dependence of the XUV and THz emission on the relative phase *δϕ*, respectively. Independent of the axial position of the gas nozzle with respect to the focus, modulations of emitted even and odd harmonics versus *δϕ* appear predominantly in phase (dotted line in Fig. 2(a)). Comparison of the two emissions (XUV and THz) reveals opposite *δϕ* dependence: while the XUV is maximized for *δϕ* ≈ *mπ*, the simultaneously emitted THz is maximized at *δϕ* ≈ (*2m* + *1*)*π/2*. This anti-correlated dependence is observed here for the first time and can be qualitatively understood by considering classical electron trajectories. According to the 3-step model, those electrons responsible for high harmonic emission follow closed trajectories that terminate in a collision with a parent ion within a half-cycle of the driving pulse. Instead, in accord with the plasma current model (PCM)^24^, THz radiation is generated from a time-varying net charge displacement of tunnel-ionized electrons in open trajectories, accumulating with each half-cycle of the driving field. This accumulation is possible only if the instantaneous drift velocity *v* is polar asymmetric and since 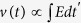, therefore it is the polar symmetric field (with *δϕ* ≈ (*2m* + *1*)*π/2*) which maximizes THz yield. The emitted power is predicted to follow sin^2^*(δϕ)* which is in close agreement with our observation (see fits in Fig. 2(b,d,f)), earlier results in ref. 24 and Eq S1 of the Supplementary Information). Good agreement of the THz yield with the fits predicted by the PCM indicate that the effects of Coulomb attraction and subsequent soft recollisions^29^ may not play a significant role in the overall THz emission process, within the demonstrated level of our experimental uncertainty. Realizing that the observed THz power follows ≈sin^2^*(δϕ),* independent of *η* (Fig. 2(b,d,f)), provides us with a convenient and an independent measure of the relative phase *δϕ* for cases of weak polar asymmetry.

In contrast to strong asymmetry, lowering *η* (=0.005) while the gas injector is positioned away from the focal plane produces negative slope in harmonics vs *δϕ* (dotted line in Fig. 2(c)). However, placement of the nozzle closer to the focus results in appearance of an additional positive slope for lower harmonic orders *q* < 28 (*ω*_*q*_ = *qω*_*0*_, see dotted lines in Fig. 2(e) and e.g.^23^,^27^). Here, it is instructive to note that emission from the short electron trajectories are known to be more efficiently phase matched further away from focus due to dipole phase contribution^30^, therefore suggesting that the two observed slopes in Fig. 2(e) potentially originate from different electron trajectories. The combined results in Fig. 2(a,c,e) clearly demonstrate that the spectra of high harmonics intricately depend on precise conditions of the non-perturbative emission process, in turn controlled by the details of the synthesized driving field (*η* and *δϕ* parameters) as well as propagation considerations. Deciphering such complexity requires experiments with a good degree of systematic control of the key parameters, thus allowing us to glean a more complete physical picture than previously attempted.

To gain further insight into the two-color-driven XUV and THz emission processes, we develop a simple analytical model based on classical electron trajectories used in the plasma current model. To this end, we broaden Corkum’s Simple Man’s model^18^ to include an intuitive and compact expression for the HHG spectrum, which is valid for arbitrary linearly polarized two-color excitation with both weak and strong polar asymmetry. As a result, our extended three-step (ETS) model captures all essential experimental details for various symmetry-broken fields and successfully explains the observed correlations of XUV and THz radiation, both processes arising out of superposed emissions from different classical electron trajectories. Furthermore, the model yields a quantitative prediction on the amplitude of the slope lines versus *δϕ* and their dependence on the intensity of the driving pulse. As is shown below, these predictions are in good agreement with our observations as well as with a broad range of independent experiments.

We consider two-color excitation pulses having a combined electric field at a fixed position along the propagation axis *E*(*t*, *δϕ*) = *A*_1_(*t*)cos(*ω*_0_*t*) + *A*_2_(*t*)cos(2*ω*_0_*t* + *δϕ*), where *A*_1_(*t*) and 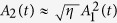 are the pulse envelope at the fundamental *ω*_*0*_, and second-harmonic 2*ω*_*0*_ frequencies with *η* denoting the fraction of power in the second-harmonic field. The local HHG electric field *E*_*H*_(*ω*, *δϕ*) is obtained by summing the contributions from all classical electron trajectories that arise during the excitation pulse:


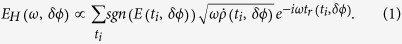


The sum is performed over all the birth times *t*_*i*_ terminating at time *t*_*r*_ in a recollision which in turn is taken to be deterministically emitting an XUV photon with energy *ħω* = *U*(*t*_*i*_, *t*_*r*_, *δϕ*) + *I*_*p*_ with *U* and *I*_*p*_being the return energy of the electrons and ionization potential of the gas, respectively. 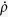 is the ionization rate, *sgn*(*E*) ensures the centro-symmetry of the medium (see Supplementary Information for additional details). Equation (1) constitutes the central result of the ETS model, providing a direct route for calculation of the spectrum of the emitted harmonics directly from the classical electron trajectories. In deriving this simple equation, therefore, we do not attempt to predict the absolute magnitude of the HHG conversion yield as we exclude the propagation effects (phase-matching) and the quantum-mechanical probabilities in the recombination process; the latter is a consequence of the classical nature of the model. The dependence of the electron transit time Δ*t* = *t*_r_ − *t*_i_ and return energy *U* together with the ionization rate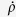 on the subcycle time structure of the driving field is shown in Fig. 3 for the cases of weak (Fig. 3(a,b)) and strong (Fig. 3(c,d)) polar asymmetries. Before highlighting the comparison of these results to the experimental findings, we would like to emphasize the motivation for resorting to a simple classical model leading to equation (1). While realizing that more rigorous approaches using quantum mechanical processes may be exploited, our simple and intuitive model using classical trajectories enables us to clearly identify the various underlying physical mechanisms manifested in the experimental observations. First, we note that within a single cycle of the excitation, the harmonic field *E*_H_(*ω*) is given as a coherent superposition of four emission contributions arising from recollision events (Fig. 3). These result from two long (L_1_, L_2_) and two short (S_1_, S_2_) electron trajectories, each with their respective amplitudes 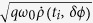 and phases *qω*_0_*t*_*r*_(*t*_*r*_, *δϕ*), determined from the tunneling rate and the classical equation of motion. The pair-wise interference of the subsequent long and short trajectories is governed by their respective differential phase terms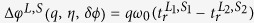. With no SH injection (*η* = *0*), Δ*φ*^*S*^ = *φ*^*L*^ = *qπ* due to an exact polar symmetry of the driving field. Deviation of *∆φ*^*L,S*^ from *qπ* due to SH injection is thus a direct consequence of the broken polar symmetry which can be controlled by the phase *δϕ* and relative amplitude 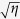 of the SH field. Numerical evaluations of *∆φ*^*L,S*^ indicate that, for a given return energy, the corresponding temporal separation between recollision events in the two half-cycles shrinks and expands for the long and short trajectories, respectively (Fig. 3).

Starting with the case of high polar asymmetry (*η* = 0.1) for *δϕ* = *mπ* (Fig. 3(c)), the extreme nonlinearity of the tunnel ionization suppresses the amplitude (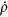) in the adjacent half cycle, thus leading to a nearly full-cycle periodicity of the HHG signal in the time domain. Fourier analysis translates this periodicity into the formation of both odd and even harmonics. At phase delays *δϕ* = (*2m* + *1*)*π/2*, the time-domain signal returns to half-cycle periodicity but with much lower amplitude (Fig. 3(d)). Thus, for strong polar asymmetry the XUV emission is predicted to peak at *δϕ* ≈ *mπ* for *all* harmonic orders, as can also be directly seen from the calculated spectral dependence on *δϕ* (Fig. 4(a)). This result is in good qualitative agreement with the measurements (Fig. 2(a)). Furthermore, the XUV dependence on the relative phase is precisely anti-correlated with the THz emission process (Fig. 2(b)), thus recovering the intuitive picture of essentially mutually exclusive nature of XUV and THz generation for the case of strong SH injection.

We now turn to the analysis of weak second harmonic injection. Using equation (1) with input of the calculated dynamic parameters from Fig. 3(a,b), one obtains the dependence of the XUV spectra on the relative phase (Fig. 4(b)). In general, the simulated spectral map reproduces the qualitative features observed in our experiment (Fig. 2(e)) as well as by other groups^20^,^21^,^23^,^27^,^28^. In particular, we note that for harmonic orders *q* ≤ 28 the calculation and experiments both show the shift in the XUV extrema with increasing *q* as a function of phase delay *δϕ*, i.e. positive values of the slopes 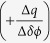. In addition to the correct dependence, the ETS model prediction of the slope of 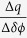 = +16 harmonics per radian is also in a remarkable agreement with the experiment (dotted lines in Figs 4(b) and 2(e) for q ≤ 28, respectively).

While the observed agreement validates the model for lower harmonics, the question remains as to why the model seemingly struggles to reproduce the experimental features seen for higher harmonic orders. As was already mentioned, the XUV spectra strongly depend on the phase matching conditions, with preference for short or long trajectories depending on the position of the gas injector. Since the ETS model does not take propagation effects into account, it is therefore unrealistic to expect a general agreement of the calculations based on conditions which might be beyond the applicability. Nonetheless, since equation (1) includes contributions from both trajectories (together with results in Fig. 2(c,e)) one anticipates that the electrons following short(long) trajectories are responsible for observed spectral features at higher(lower) harmonics and the two dominant regions are separated by a spectral band where both contributions are comparable (e.g. 28 < q < 32 in Fig. 2(e) for particular phase-matching conditions). To elaborate on this we turn our attention to the amplitudes 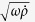
of these emission events, the temporal structures of which already played a decisive role in breaking half-cycle periodicity for strong polar asymmetry (Fig. 3(c,d)). Instead, for weak polar asymmetry the electrons which undergo long trajectory recollision events are generated at times before the peak of the electric field (Fig. 3(a,b)). This fact leads us to conclude that the highly nonlinear tunneling ionization rate favors these events over the short trajectories that are in contrast born at later times. To check the validity of these observations, we calculate the spectral dependence of the amplitudes 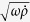 of short and long trajectories for *δϕ* = 0 and *π*/2, depicted in Fig. 3(e,f) with blue and red lines, respectively. At low harmonics, the total amplitude is dominated by the contribution from the long trajectories, while long and short trajectories assume comparable amplitudes as harmonics approach the cut-off region. This explains why all the experiments (including ours) show the positive slope 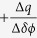 at lower harmonics, corresponding to long trajectories. In contrast, at energies approaching the cut-off harmonics, one expects a more complex behavior resulting from an interference of all 4 trajectories. This is indeed the case in the experiments where there is no additional selectivity that distinguishes short and long trajectories. In practice, such selectivity can be implemented by exploiting the far-field divergence of the XUV emission leading to a spatial separation of contributions from long and short trajectories^26^ or preferential phase-matching^31^.

Finally, to further elucidate the roles of amplitude and phase of the trajectories and to further clarify the complexity of the spectrum for harmonics, we cast the expression in equation (1) into an even simpler form. For a square driving pulse containing *N* cycles at fundamental frequency, an approximation of 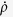
scaling as *|E|*^*Q*^ and consideration of the dominant birth times to be near the peaks of each cycle, produces an HHG spectrum:


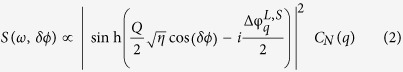


where *C*_*N*_(*q*) = sin^2^(*Nπq*)/sin^2^(*πq*) is a comb function, 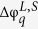 are the differential phase terms defined earlier and *Q* ≈ *7*–*9* for typical laser intensities involved. A plot of this simple expression for long and short trajectories during weak polar asymmetry (Fig. 4(c,d)) reproduces all the essential features of the more exact calculation (Fig. 4(b)) and experimental observations (Fig. 2(c,e)).The calculation also faithfully reproduces XUV spectra for strong polar asymmetry (not shown). Furthermore, the differential phase terms in equation (2) are determined to follow the following empirical functions: 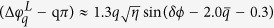, and 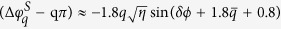, where 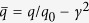 with *q*_*0*_ = *2U*_*p*_*/ħω*_*0*_, *U*_*P*_ being the ponderomotive energy and 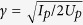 denoting the Keldysh parameter (see also Supplementary Information). The peaks of harmonics occur at 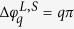 corresponding to a stationary phase (=*mπ*, *m* integer) of the sine functions. Evaluated at these stationary points, the two expressions yield opposite signs of slopes 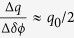 and 
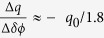
 for long and short trajectories, respectively (also can be directly seen in Fig. 4(c,d)). Furthermore, these slopes are found to depend on the peak intensity and the center wavelength of the fundamental field: 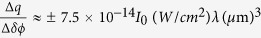 (harmonics per radian) (see Fig. S2 in Supplementary Information). Evaluating this result for *λ* = 0.8 μm, we find 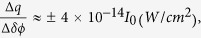 which correspond to slopes of ≈ ±16 harmonics/radian for long and short trajectories, based on our estimated focal intensity of *I*_*0*_ = 4 × 10^14^ W/cm^2^. Both in sign and magnitude, these slopes are in an excellent agreement with our experimental findings (Fig. 2(e)). Notably, in addition to the agreement with our results, the prediction of the ETS model of the amplitudes of the slopes is also in a very good agreement with a rather general set of reported experimental observations^20^,^21^,^23^,^27^,^28^. Finally, we estimate reduction in the peak intensity of our fundamental pulse by a factor 3–4 when translating the gas injector away from the focal plane to a new position (conditions in Fig. 2(c)). We observe a commensurate reduction in the slope from 16 ± 0.3 harmonics/rad (Fig. 2(e)) to a value of 5 ± 0.3 harmonics/rad (Fig. 2(c)). The overall drop in the slope value by a factor of 3.2 ± 0.6 during an estimated 3–4 times drop in the intensity shows very good agreement with the predictions of the ETS model (see previous paragraph and Supplementary Fig. S2).

In conclusion, we report detailed investigation of the XUV emission spectra from argon gas when driven by intense femtosecond pulses while being subjected to a transient optical bias induced by the phase-locked second harmonic pulse replica. By varying the intensity ratio (*η*) and the relative phase (*δϕ*) of the co-polarized second harmonic pulses we are able to systematically study the resulting spectra of the XUV emission and the yields of the simultaneously-emitted THz radiation. For strong injection of the second harmonic (10% of the fundamental intensity or strong polar asymmetry) we find that XUV and THz radiation are anti-correlated in their dependence on the relative phase *δϕ.* Instead, for weak polar asymmetry we find that while THz dependence on *δϕ* remains unchanged, the XUV spectra show remarkably complex structure, which, while also dependent on the phase-matching considerations, reveals two distinct slopes, defined by a phase slip 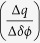 between the adjacent harmonic orders q. To explain these features we devised an extended three-step model (ETS) based on the classical electron trajectories. With the gained physical insight on the origin of XUV emission to arise from interference of XUV bursts from multiple recollision events of short and long trajectories within a single cycle of the driving pulse, we are able to provide qualitative explanations for all the experimentally observed features. Very good qualitative agreement between experimental results and the model points to the robustness of the semi-classical approach of the original 3-step model of high harmonic generation. Furthermore, quantitative prediction of the ETS model for the sign and magnitude of the slopes 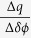 has been verified in our experiments and found to also reproduce a wide array of previously published results by other groups. Curiously, dependence of these slopes on the intensity of the fundamental pulse might suggest future routes for XUV pulse, as verified by our experiments, compression schemes with control of the spectral phase of harmonics, known as attochirp^21^.

Finally, gained insight into the contributions of short and long trajectories in cases of weak and strong polar asymmetry in the driving two-color field allows us to draw further conclusions about the relation of the XUV and THz emission processes. It is clear from the nature of the non-trivial phase shifts with increasing harmonic orders 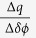 that in case of weak polar asymmetry no absolute phase relationship between the two processes can be established *a priori*. In turn, XUV emission resulting from strong polar asymmetry has shown a clear phase dependence which is anti-correlated in the relative phase parameter with the production of the THz. It is only from a careful comparison of these two regimes that precise conclusions on the phase-dependence of all the processes can be drawn. Thus our experiments and the ETS model provide a unified picture of XUV and THz emission mechanisms within the framework of classical trajectories and arbitrary degree of polar asymmetry in the driving field. Furthermore, the agreement of the observed correlation features in the THz and XUV emission with our theory suggests that the two processes have the same physical origin, only with different electron trajectories. Together with the high degree of control, perhaps it is sources like these that in the future could provide ultrabroad emission in the entire THz-XUV frequency window with potential technological and scientific applications.

## Methods

### Experimental Details

**Figure 1:**
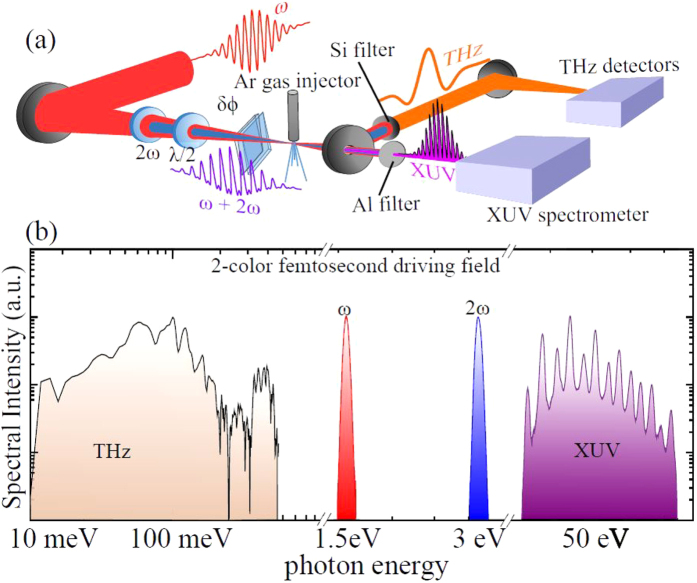
Coherent ultrabroadband emission from 2-color optical pulses focused into an argon gas jet. (**a**) Schematic of a two-color femtosecond laser system which output is focused onto a gas jet to produce ultra-broadband spectra of coherent electromagnetic radiation capable of spanning an astounding 3 or more orders of magnitude in frequency/energy; labels “2ω”, “λ/2”, “δϕ”, “Al filter” and “Si filter” designate second harmonic generation crystal, zero-order half-wave plate, pair of glass wedges for the control of the relative phase between the fundamental and second harmonic components, aluminum filter (to block excitation while transmit XUV) and silicon filter (to block excitation while transmit THz), respectively. (**b**) Emission from argon gas, driven by focused 40-fs pulses at 375 THz and their second harmonic (“ω + 2ω”), is depicted in a double-logarithmic plot where “THz” emission extends from 1 THz to over 90 THz (1 THz = 10^12^ Hz, corresponding to 3meV) and the “XUV” emission spans from 1 to 12 PHz (1 PHz = 1 × 10^15^ Hz), i.e. from 4 to 50 eV in photon energy. THz spectrum was measured by broadband autocorrelation, performed with in nitrogen gas and outside the vacuum chamber. Nonetheless, it schematically shows the potential spectral coverage of such source at low frequencies. At the same time, modulations of the extreme UV emission are due to the interference of XUV bursts, manipulated by the temporal sub-cycle structure of the two-color field (see text for additional details). The bandwidth is mainly limited by the choice of the noble gas and the operating parameters of our XUV spectrometer. Spectral intensities of different emission components are normalized independently.

The experimental setup for synchronous generation and detection of XUV and THz radiation is shown schematically in Fig. 1. A train of 1 kHz, 40 fs pulses with a center wavelength of 800 nm produces high harmonics when focused onto the output of an argon gas injection nozzle with peak intensity of (4–5) × 10^14^ W/*cm*^2^. Our experiments are done at high gas pressures of up to 1000 Torr and the laser beam is loosely focused under the gas injection nozzle with a diameter of 0.3 mm. Under these conditions, both long and short trajectories can be phase-matched. Preferential generation of short trajectories can be selected by locating the nozzle after the focus^31^.

The SH of fundamental field at angular frequency *ω*_0_ is generated in a 150 *μm* type-I BBO crystal, the orientation and position of which control the conversion efficiency of SH in the range of 0.5% to approximately 10%. Parallel polarization of the two fields is ensured by an ultra-thin, true zero-order half-wave plate for the fundamental wavelength. As expected from symmetry arguments and confirmed by measurements, this results in linearly polarized THz and XUV emission. A pair of glass wedges controls the relative phase *δϕ* and hence the degree of asymmetry between two pulses with an overall accuracy ≈*π*/5 that is independently calibrated in a separate experiment^32^ (also see Supplementary Information). All of these optical components are placed in a low background pressure chamber to ensure minimal phase-slip between the fundamental and SH pulses. An off-axis parabolic mirror is located after the gas nozzle and serves to collimate the THz field out of the vacuum chamber through a silicon viewport. This window also acts as an optical filter to remove the excitation light. A 500 *μm* diameter central hole in the parabolic mirror transmits the low-divergence XUV beam, which then passes through a 200-nm thick aluminum filter and is routed to a grazing-incidence XUV spectrometer (McPherson Inc.), as shown in Fig. 1. Terahertz emission is field-resolved with electro-optic sampling (500-*μm* thick (110) ZnTe detector) and direct detected by a pyroelectric sensor. Comparison of measured signals by both of these techniques verifies that THz yield is frequency independent, resulting in the overall scaling of the magnitude of the emission process with the variation of the relative phase *δϕ*. Polarization states of the XUV and THz pulses are ensured using polarization-dependent anisotropy of the grating reflectivity and a broadband polarizer, respectively, both with polarization contrast better than 10 to 1.

## Additional Information

**How to cite this article**: Gragossian, A. *et al*. Classical trajectories in polar-asymmetric laser fields: Synchronous THz and XUV emission. *Sci. Rep.* **6**, 34973; doi: 10.1038/srep34973 (2016).

## Supplementary Material

Supplementary Information

## Figures and Tables

**Figure 2:**
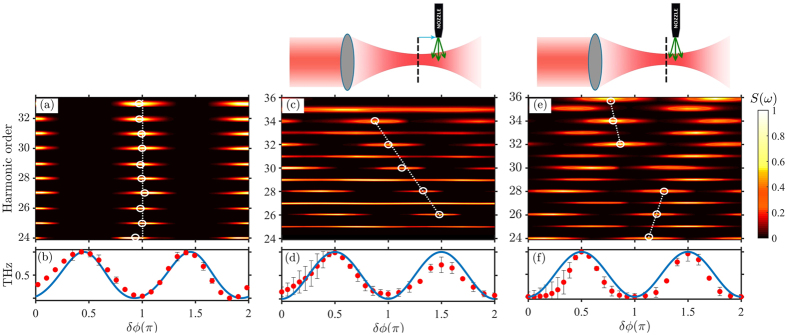
Characterization of simultaneous emission of XUV and THz radiation from argon gas, driven by 2-color pulses with variable degree of polar asymmetry. Dependence of the spectra of high harmonic emission (ω_q_ = qω_0_ with harmonic order q, top row) and THz yield (bottom row) on the relative phase between the fundamental ω_0_ and second harmonic 2ω_0_ fields (δϕ) measured at conditions of strong (η = 0.1, panel (**a**,**b**)) and weak (η = 0.005, panel (**c**–**f**)) polar asymmetry. XUV spectra also show dependence on the position of the gas nozzle with respect to the focal plane of the excitation (sketched above, see text for details). Positive and native slopes (Δq/(Δδϕ) in the XUV emission maps are depicted by dashed lines, connecting maxima of the neighboring harmonic orders (open circles). Measured normalized average power of the simultaneously emitted THz pulses (red filled circles) is shown versus δϕ for strong (**b**) and weak (**c**,**e**) polar asymmetries. Blue line is an exact calculation from the plasma current model (also ETS model here). Opposite dependence of THz and XUV emission in (**a**,**b**) on δϕ is explained by the different classical electron trajectories, participating in the respective emission mechanisms, as explained in the text.

**Figure 3:**
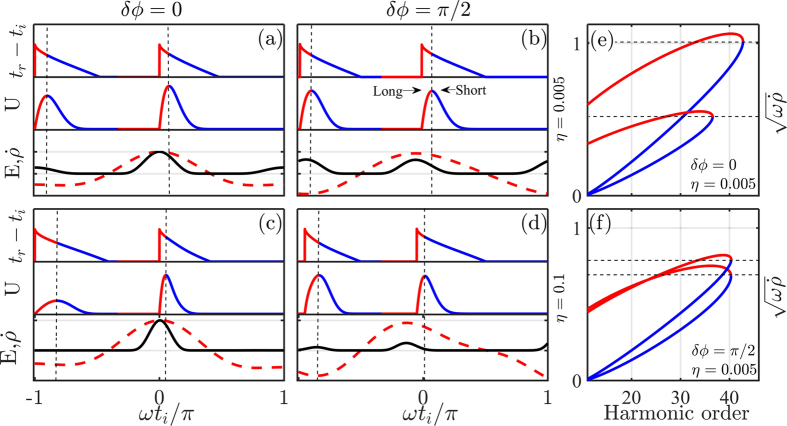
Calculated electron trajectories on a sub-cycle timescale of the driving electric field within the ETS model. The electron transit time (Δt = t_r_ − t_i_) and return kinetic energy U, the electric field E (dotted red line) and ionization rate 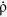 (black line) are depicted versus the ionization time (ω_0_t_i_ for two extreme relative phase delays for η = 0.005 (**a**,**b**) and η = 0.1 (**c**,**d**). (**e**,**f**) show the relative harmonic amplitudes 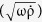 generated from short (blue) and long (red) trajectories for η = 0.005 and δϕ = 0 and δϕ = π/2, respectively. The black doted lines indicate the maximum harmonic energy.

**Figure 4:**
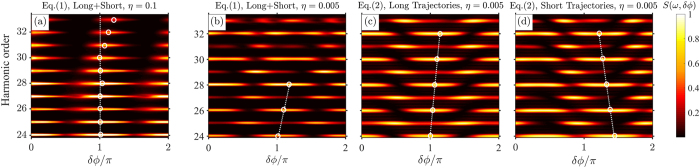
Calculated spectra of the XUV emission from the ETS theory. Normalized XUV spectra S(ω, δϕ) = |E_H_|^2^ are calculated from equation (1) for cases of strong (η = 0.1, panel a) and weak (η = 0.005, panel b) polar asymmetry of the driving two-color field. For η = 0.005, the contributions from short and long trajectories are separated in equation (2) with Q = 8 and N = 4 cycles (see text for details). Indicated slopes are in a very good agreement with the measurements, presented in Fig. 2.
