# Supplementary material for: Classical trajectories in polar-asymmetric laser fields: Synchronous THz and XUV emission
Source: Sci Rep. 2016 Oct 19;6:34973. doi: 10.1038/srep34973 (PMC5069465; doi:10.1038/srep34973)
Supplement: Supplementary Information [file srep34973-s1.pdf]

Supplementary Information: Classical trajectories in polar-asymmetric laser fields: Synchronous THz and XUV emission

Aram Gragossian[1], Denis V. Seletskiy[1,2], and Mansoor Sheik-Bahae[1*]

[1]University of New Mexico, Physics and Astronomy Dept., 1919 Lomas Blvd. NE, Albuquerque, NM 87131, USA

[2]Department of Physics and Center for Applied Photonics, University of Konstanz, 78457 Konstanz, Germany

Classical Trajectories:

The first step in both THz and XUV generation by short laser pulses in gaseous media is the laser induced ionization process. As described by Keldysh[1], the dominant ionization mechanism depends on the ionization potential (I_P) of the target gas and the optical field parameter given by the ponderomotive energy ($U_p = e^2E_0^2/4m_e\omega_0^2$,m_e and e are electron mass and charge respectively and E_0 is the peak electric field amplitude in each cycle). The regime for which the Keldysh parameter $\gamma = \sqrt{I_p/2U_p}$ < 1 is where tunneling ionization is dominant while multi-photon ionization takes over when γ > 1. Using intense femtosecond laser pulses it is routinely possible to drive light-matter interaction into the tunneling regime, where the time evolution of the ionized electron density ρ is given by $\dot{\rho} = [\rho_0 - \rho(t)]w(|E(t)|)$ where ρ_0 is the initial (unionized) atomic density, and $w(t) = 4\omega_a\left(\frac{E_a}{E(t)}\right)exp\left(-\frac{2}{3}\frac{E_a}{E(t)}\right)$ is the tunneling rate for hydrogen-like atoms in a semi-static electric field[2].$E_a = 5.14\times10^9\, V/cm$ and $\omega_a = 4.134\times 10^{16}s^{-1}$ is the atomic frequency.

The second step following an ionization event (at time t_i) is the acceleration of electrons subject to time varying electric field $E(t)$. For linearly polarized fields (along x-axis) this is simply given by the classical equation of motion $m_e\ddot{x} = -eE$, where dotted notation stands for the temporal derivative. In the two-color excitation, the resulting polar asymmetric field is given by$E(t,\delta\phi) = A_1(t)cos(\omega_0 t) + A_2(t)cos(2\omega_0 t + \delta\phi)$ where $A_1(t)$ and $A_2(t) \approx \sqrt{\eta}\,A_1^2(t)$ are the pulse envelope at the fundamental ω_0, and second-harmonic $2\omega_0$ frequencies with η denoting the fraction of power in the second-harmonic field.

Here is where the THz and XUV generation part ways: Based on the macroscopic plasma current model[3,4], the THz emission is due to accelerating charges radiating subject to a two-color field.

Ignoring propagation (phase-matching) effects, and in the absence of collisions, the local THz field can be expressed as the time derivative of the current density ($E_{THz}(t) \propto \dot{J}$) which in turn is given by:

$$J(t,\delta\phi) \approx \int_{-\infty}^{t} e\dot{\rho}(t_i,\delta\phi)v_d(t,t_i,\delta\phi)dt_i \qquad \text{(S1)}$$

*e-mail: msb@unm.edu

where $v_d(t, t_i, \delta\phi) = \dot{x} = (-e/m_e)\int_{t_i}^{t} E(t', \delta\phi)dt'$ is the instantaneous electron drift velocity of electrons ionized at time t_i as obtained from the classical equation of motion. Further simplification leads to $E_{THz}(t) \approx -\frac{e^2}{m_e}\rho(t, \delta\phi)E(t, \delta\phi)$. At low level injection of $2\omega_0$ (η<<1), it was shown that the THz component is fairly approximated by $E_{THz}(t) \propto f(|A_1(t)|/E_a)A_2(t)\sin(\delta\phi)$[3], with $f(x) = x^{-1/2}\exp(-\frac{2}{3x} - 3x)$ and E_a=5.14×10^9 V/cm (atomic field) thus leading to a $sin^2(\delta\phi)$ dependence in the detected THz power[4–7]. Numerical evaluation of THz verifies this dependence and only small deviations from $sin^2(\delta\phi)$ is predicted at high intensities (≈0.04π deviation for 4×10^{14} W/cm^2). It is important to note that due to highly nonlinear nature of the ionization rate, equation (S1) contains not only a subharmonic term (thus THz), but also higher harmonics of the incident fields. These so-called "drift harmonics" are however much weaker than those that will be generated by recombination (recollision) of the ionized electrons with the parent ion -which is the last step of the 3-step model. Finally, we mention that the role of Coulomb interactions and subsequent soft recollisions (see Ref. 28 of the main text) is also expected to show deviation from the predicted $sin^2(\delta\phi)$ dependence. Improvement of the absolute precision in determination of the $\delta\phi$ is required to investigate this and other predicted deviations in more detail.

As Corkum eloquently explained, the non-perturbative nature as well as the cut-off energy of high-harmonic generation (HHG) can be captured by a 3-step classical model that contains the 2-steps mentioned above (namely ionization and acceleration) being followed by a third-step which is the recombination. In this picture, a fraction of electrons, depending on their birth time, can acquire sufficient kinetic energy to trigger high-frequency photon emission upon recombination with the parent ion. This simple, insightful model was confirmed by a quantum-mechanical approach in the strong-field-approximation[8,9] followed by a detailed conversion efficiency analysis[10,11]. A rigorous description of the ionization and the ensuing electronic wavepacket dynamics was performed by numerical evaluation of time-dependent Schrödinger equation (TDSE)[12–14].

Full quantum mechanical description based on numerical solution of TDSE have been also used to verify the validity of the plasma current model in the classical limit[7,15,16].

We emphasize that this *intuitive* treatment of HHG is based on classical trajectories. By its very nature, it cannot be rigorous as it makes a quantum leap (literally) by assuming that each re-collision produces a photon in a deterministic way. Not to mention that the tunneling ionization itself is quantum mechanical in nature nor the fact that the theory ignores the quantum nature of the liberated electrons, namely the spreading of their wavefunctions during the transit time.

In what follows, we outline additional details on our extension of this classical 3-step model, allowing us to compute and interpret harmonic emission spectra following 2-color excitation. Here, we are not concerned with the absolute magnitudes, conversion efficiency, or propagation evolution including the phase-matching requirements. Our primary objective is to elucidate the critical role of the polar asymmetry of the excitation field, i.e. to provide understanding of the effect of SH power (η) and its relative phase delay ($\delta\phi$) with respect to the fundamental on the spectral features of the high harmonic emission.

Extended Three-Step Model:

After an ionizing event, the transverse electron trajectories $x(t_i, t, \delta\phi)$ at time t are obtained from the classical equation of motion ($m_e\ddot{x} = -eE$) given a birth time t_i and zero initial momentum, thus assuming $\dot{x}(t_i, t_i, \delta\phi) = 0$ and $x(t_i, t_i, \delta\phi) = 0$ as initial conditions. As in the one-color excitation, the return times (t_r) for each trajectory are obtained from the solution of $x(t_i, t_r, \delta\phi) = 0$, with a return kinetic energy $(t_i, t_r, \delta\phi) = m_e\dot{x}(t_i, t_r, \delta\phi)^2/2 \equiv U_pF(t_i, t_r, \delta\phi)$. Exploiting the well-known relationship between the field amplitude and the photon number for a given carrier frequency, we take the spectral amplitude of the emitted harmonics to vary as $|E_H| \propto \sqrt{\hbar\omega\dot{\rho}}$ where $\dot{\rho}(t_i)$is the ionization rate at a given birth time (t_i) for electron trajectories having return energy U that satisfies $U(t_i, t_r, \delta\phi) + I_p = \hbar\omega$. Next, we assign a relative spectral phase ωt_r to emission from such a trajectory; *this signifies the distinct re-collision (arrival) times of each trajectory bunch in the time domain*. $\dot{\rho}$ represents the instantaneous ionization rate most commonly attributed to tunneling. The sign function *sgn(E)* in equation (1) of the main text is introduced to ensure the centro-symmetry of the medium, i.e. implying that the radiation resulting from right and left trajectories should have opposite polarity. The total electric field at a given frequency ω $(=q\omega_0)$ is then the coherent sum (or interference) of emissions from all the trajectories during the pulse as given by equation (1) in the main text. For simplicity, equation (*1*) is derived assuming uniform spectral density of the trajectories, i.e. $\left|\frac{\partial U}{\partial t_i}\right| = constant$, which is a fair approximation for the plateau harmonics.

The strength of the analytical approach is in the physical insight that can be gained in a straight forward way. For instance, the spectral features of $|E_H(\omega)|^2$ in equation (1) can be understood to result from the interference of harmonic radiations coming from subsequent half-cycles of the driving field. Furthermore, the emission can be understood to result from pairs of long and short classical trajectories in each optical cycle of the fundamental, for a total of four, as denoted by L_1, L_2, S_1 and S_2, respectively. The pair-wise interference of the subsequent long and short trajectories is therefore governed by their respective difference in the phase: $\Delta\varphi^{L,S}(q, \eta, \delta\phi) = q\omega_0(t_r^{L_1,S_1} - t_r^{L_2,S_2})$. . With no injection ($\eta = 0$) of the second harmonic field, $\Delta\varphi^S = \Delta\varphi^L = q\pi$ due to an exact polar symmetry of the driving field. Deviation of $\Delta\varphi^{L,S}$ from $q\pi$ due to SH injection is thus a direct consequence of the broken polar symmetry, which can be modulated by the phase $\delta\phi$ and relative amplitude $\sqrt{\eta}$ of the SH field. Numerical evaluation of $\Delta\varphi^{L,S}$ under finite SH injection indicates that for a given return energy, the corresponding temporal separation between recollision events in the two half-cycles shrinks and expands for the long and short trajectories, respectively. This behavior is depicted in Fig. S1 where differential phase $\Delta\varphi^{L,S}(q, \eta, \delta\phi) - q\pi$ terms for both short and long trajectories are plotted versus $\delta\phi$ and a normalized return energy defined as $\bar{q} = q/q_0 - \gamma^2$ where $q_0 = 2U_p/\hbar\omega_0$.

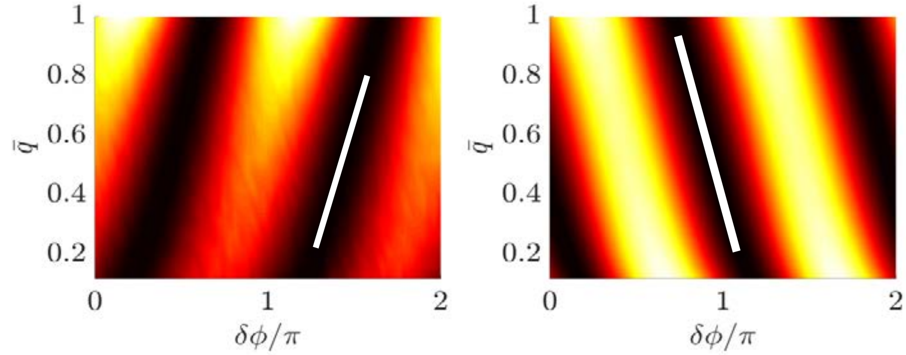

Fig. S1: Calculated differential phase $\Delta\varphi^{L,S}(q,\eta,\delta\phi) - q\pi$ for both short and long trajectories are plotted versus $\delta\phi$ and a normalized return energy given by $\bar{q} = q/q_0$. The obtained slopes (of the solid titled lines) are in universal agreement with the measurements with our measurements as well as other results (see text for details).

The solid white lines in Fig. S1 map the loci of vanishing differential phase in each case with slopes $\frac{\Delta q}{\Delta\delta\phi} \approx +q_0/2$ and $\frac{\Delta q}{\Delta\delta\phi} \approx -q_0/1.8$ for long and short trajectories respectively. Substituting q_0 (see main text), these slopes are approximated as $\frac{\Delta q}{\Delta\delta\phi} \approx \pm 7.5 \times 10^{-14} I_0(W/cm^2)\lambda(\mu\mathrm{m})^3$ (harmonics per radian). Taking $\lambda = 0.8$ μm, we obtain $\frac{\Delta q}{\Delta\delta\phi} \approx \pm 3.84 \times 10^{-14} I_0(W/cm^2)$. These lines are plotted in Fig. S2 for intensities I_0 ranging from $(2-8)\times10^{14}$ W/cm^2. With $I_0 \approx 4\times10^{14}$ W/cm^2, which is the intensity used in nearly all the experiments[17–21], we obtain a slope $\approx \pm 16$ harmonics/radian. All the reported experiments (including ours) exhibit a slope close to this value indicating that the intensities involved must have been around 4-5$\times10^{14}$ W/cm^2 which is indeed the case. We attempted to validate this dependence experimentally by increasing the intensity of the fundamental field to $\approx 8\times10^{14}$ W/cm^2 but the slope remained nearly the same. Interestingly, equation (1) also reproduces this result which is simply a manifestation of ground-state depletion, which favors low intensities for pulses 40 fs and longer. To experimentally observe this dependence, we believe short pulses (20 fs and shorter) should be used to avoid the ground-state depletion.

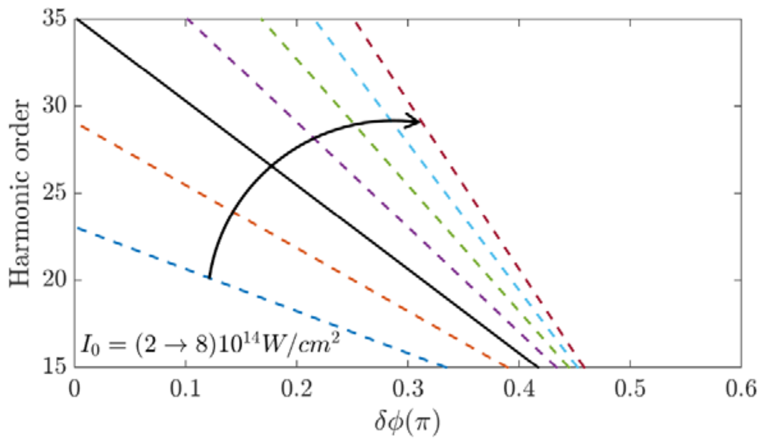

Fig. S2: Calculated variation of the differential phase for each harmonic resulting from short trajectories versus the asymmetry $\delta\phi$, parametrized by the varying intensity I_0 of the fundamental field. Solid line exhibiting a negative slope of 16 harmonics per radians corresponds to the intensity used in our experiments. Arrow indicates the direction of increasing intensity through the range of values displayed in the bottom left corner.

Calibration of the Relative Phase:

In addition to the comparison of the slopes, our experiments also make a reference to the dependence on the absolute value of the $\delta\phi$ parameter. Given the generally non-perturbative character of both THz and XUV generation processes, we first have to understand how one of these processes depends on the $\delta\phi$. To accomplish this, we performed calibration experiment where THz dependence was measured against a known signal. In our case, the reference was derived from a cascaded second-order nonlinearity, where two SHG crystals (Type I BBO) where put in sequence. The first crystal stands for the SH crystal used in the main experiment while the

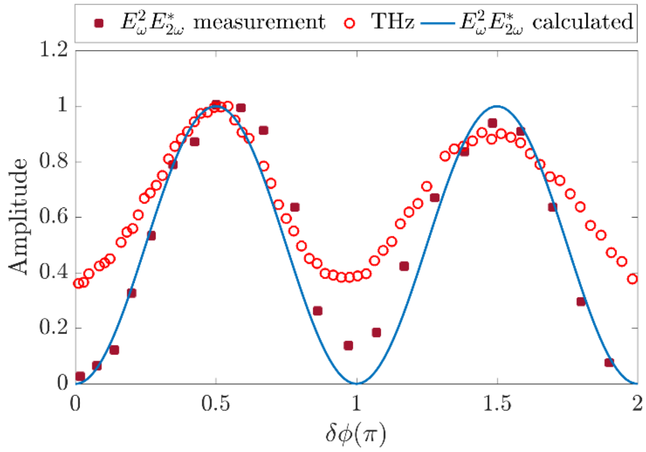

Fig. S3: Measurement of the dependence of the THz generation (open circles) and reference experiment based on perturbative nonlinear optics (closed squares, see text) on the $\delta\phi$ parameter and versus the perturbative theory (solid line). The reference signal is used to verify the dependence of the THz generation mechanism on the absolute value of $\delta\phi$.

second one is placed to mimic the THz generation process. In the case of the $\chi^{(2)}$: $\chi^{(2)}$ experiment, the relative phase $\delta\phi$ controls whether the generated SH signal in the second crystal is in- or out-of-phase[22] with the original "driving" SH. The relative phase is changed by the pair of glass wedges. Figure S3 shows the result of the measurement where THz and reference signals show the dependence on $\delta\phi$, the reference in a very good agreement with the perturbative theory. This measurement allows us to conclude that THz emission is predominantly suppressed at $\delta\phi = \pi$ and allows us to make further conclusions on the dependence of the XUV emission on the relative phase parameter, as detailed in the main text. Our current accuracy on the knowledge of the $\delta\phi$ parameter is $\approx \pi/5$, which is enough to support the main claims in the main text.

Supplementary References:

1. Keldysh, L. V. Ionization in the field of a string electromagnetic wave. *J. Exp. Theor. Phys.* **20,** 1307–1314 (1965).
2. L D Landau, E. M. L. *Quantum Mechanics*. (Pergamon, 1977).
3. Kim, K. Y., Taylor, A. J., Glownia, J. H. & Rodriguez, G. Coherent control of terahertz supercontinuum generation in ultrafast laser–gas interactions. *Nat. Photonics* **2,** 605–609 (2008).
4. Babushkin, I. *et al.* Ultrafast spatiotemporal dynamics of terahertz generation by ionizing two-color femtosecond pulses in gases. *Phys. Rev. Lett.* **105,** 1–4 (2010).
5. Kim, K.-Y. Generation of coherent terahertz radiation in ultrafast laser-gas interactions. *Phys. Plasmas* **16,** 056706 (2009).
6. Li, C.-Y., Seletskiy, D. V., Yang, Z. & Sheik-Bahae, M. Broadband field-resolved terahertz detection via laser induced air plasma with controlled optical bias. *Opt. Express* **23,** 11436 (2015).
7. Xie, X., Dai, J. & Zhang, X.-C. Coherent Control of THz Wave Generation in Ambient Air. *Phys. Rev. Lett.* **96,** 075005 (2006).
8. Lewenstein, M., Balcou, P., Ivanov, M., L'Huillier, A. & Corkum, P. Theory of high-harmonic generation by low-frequency laser fields. *Phys. Rev. A* **49,** 2117–2132 (1994).
9. Brugnera, L. *et al.* Trajectory Selection in High Harmonic Generation by Controlling the Phase between Orthogonal Two-Color Fields. *Phys. Rev. Lett.* **107,** 153902 (2011).
10. Falcão-Filho, E. L., Gkortsas, M., Gordon, A. & Kärtner, F. X. Analytic scaling analysis of high harmonic generation conversion efficiency. *Opt. Express* **17,** 11217–29 (2009).
11. Falcão-Filho, E. L. *et al.* Scaling of high-order harmonic efficiencies with visible wavelength drivers: A route to efficient extreme ultraviolet sources. *Appl. Phys. Lett.* **97,** 061107 (2010).
12. Krause, J., Schafer, K. & Kulander, K. Calculation of photoemission from atoms subject to intense laser fields. *Phys. Rev. A* **45,** 4998–5010 (1992).
13. Sandner, W., Figueira, C., Faria, D. M. & Do, M. Time profile of harmonic generation. *Phys. Rev. A* **55,** 3961–3963 (1997).
14. Bauer, D. & Koval, P. Qprop: A Schrödinger-solver for intense laser–atom interaction. *Comput. Phys. Commun.* **174,** 396–421 (2006).

15. Dai, J., Karpowicz, N. & Zhang, X.-C. Coherent Polarization Control of Terahertz Waves Generated from Two-Color Laser-Induced Gas Plasma. *Phys. Rev. Lett.* **103,** 1–4 (2009).

16. Cook, D. J. & Hochstrasser, R. M. Intense terahertz pulses by four-wave rectification in air. *Opt. Lett.* **25,** 1210 (2000).

17. Dudovich, N. *et al.* Measuring and controlling the birth of attosecond XUV pulses. *Nat. Phys.* **2,** 781–786 (2006).

18. Dahlström, J. M., L'Huillier, A & Mauritsson, J. Quantum mechanical approach to probing the birth of attosecond pulses using a two-colour field. *J. Phys. B At. Mol. Opt. Phys.* **44,** 095602 (2011).

19. Zhang, D. *et al.* Synchronizing Terahertz Wave Generation with Attosecond Bursts. *Phys. Rev. Lett.* **109,** 243002 (2012).

20. Lü, Z. *et al.* Attosecond synchronization of terahertz wave and high-harmonics. *J. Phys. B At. Mol. Opt. Phys.* **46,** 155602 (2013).

21. Brugnera, L. *et al.* Enhancement of high harmonics generated by field steering of electrons in a two-color orthogonally polarized laser field. *Opt. Lett.* **35,** 3994–6 (2010).

22. Chudinov, A. N., Kapitzky, Y. E., Shulginov, A. A. & Zel'dovich, B. Y. Interferometric phase measurements of average field cube $E_{\omega}^{2} E_{2\omega}^{*}$. *Opt. Quantum Electron.* **23,** 1055–1060 (1991).
